# Lack of association between AKAP2 and the susceptibility of adolescent idiopathic scoliosis in the Chinese population

**DOI:** 10.1186/s12891-017-1731-x

**Published:** 2017-08-24

**Authors:** Leilei Xu, Chao Xia, Weiguo Zhu, Zhenhua Feng, Xiaodong Qin, Weixiang Sun, Yong Qiu, Zezhang Zhu

**Affiliations:** Department of Spine Surgery, the Affiliated Drum Tower Hospital of Nanjing University Medical School, Zhongshan Road 321, Nanjing, 210008 China

**Keywords:** Adolescent idiopathic scoliosis, AKAP2, Mutation, Polymorphism

## Abstract

**Background:**

Adolescent idiopathic scoliosis (AIS) is a well characterized spinal deformity that affects millions of children world-wide. The role of genetic factor in the development of AIS has been of great interest, since obvious hereditary trend has been observed in AIS families. In a recent study of Chinese population, a novel mutation of AKAP2 was observed in a family with AIS, which was believed to play a role in the aetiopathogenesis of AIS. The purpose of this study was to investigate whether genetic variants of AKAP2 are associated with the susceptibility of AIS in Chinese population.

**Methods:**

SNV c.2645A > C of AKAP2 was genotyped in 1254 AIS patients and 1232 normal controls using allelic-specific multiple ligase detection reactions. SNPs located within 5′ untranslated regions (UTR) and 3′ UTR of AKAP2 gene were selected using Haploview (v2.6). The GWAS database composed of 961 AIS patients and 1499 controls was referred to for the genotyping information. Relative mRNA expression of AKAP2 in peripheral blood was analyzed for 33 patients and 18 age-matched controls. Comparison between the cases and controls were performed using the Student’s t test. PLINK (v1.90) was used to calculate the association of each SNP with the disease by Cochran-Armitage trend test.

**Results:**

All the patients and the controls presented a genotype of AA in c.2645A > C of AKAP2, and there was no case of mutation in any subject. A total of 116 SNPs covering AKAP2 were analyzed, and none of these SNPs was found to have significantly different allele frequency between the cases and the controls. The mRNA expression of AKAP2 in patients was comparable with that in the controls (1.9 ± 0.8 vs. 1.8 ± 0.7, *p* = 0.66).

**Conclusions:**

Our large-scale replication study of the variants in AKAP2 gene did not support its association with the susceptibility of AIS in the Chinese population. In future study, functional studies of the previously reported rare variant are warranted to clarify whether the variant can regulate the expression of AKAP2. The whole AKAP2 gene can be sequenced in larger AIS cohorts to identify potentially missing mutations.

## Background

Adolescent idiopathic scoliosis (AIS) is a well characterized spinal deformity that affects millions of children world-widely [1]. As a condition exclusive to human beings, the pathogenesis of AIS has been widely investigated in the past decades [2–6]. Among the multiple proposed factors, the role of genetic factor in the development of AIS has been of great interest, since obvious hereditary trend has been observed in AIS families [7–9]. Large population studies showed that 11% of the first-degree relatives of AIS patients could present scoliosis [9]. Moreover, AIS Twins showed a significantly higher concordance rate for the disease as compared with those reported in the first-degree relatives [10]. Despite consensus on the familial nature of AIS, the mode of inheritance has been on debate. Different inheritance patterns has been proposed, including an autosomal dominant, X-linked, and multifactorial pattern [8, 11].

To explore the genetic background of AIS, Miller et al. firstly applied genetic linkage analysis was to familial patients, which unveiled several candidate regions [12]. Later, using genetic association study, a number of susceptible genes were reported for AIS, such as ESR1, NTF3, and CDH13 [13–15]. Recently, genome-wide association studies (GWASs) conducted revealed a few more susceptible loci of AIS in the Caucasian, the Japanese and the Chinese populations [16–20]. Though a meta-analysis of the Caucasian and the Japanese GWAS dataset, Wise et al. [21] reported a functional variant in PAX1 was significantly associated with AIS. Nevertheless, the common variants reported by these association studies can only explain limited variation of AIS, since they all confer a small odds ratio to the risk of the disease.

Compared with common variants, rare variants are generally thought to exert a greater influence on the risk of inherited disease [22]. For AIS, variants of smaller effect have yet to be found, which could contribute substantially to the missing heritability [22]. With the development of sequencing technologies and reduction in sequencing cost, large-scale sequencing studies has been performed to detect rare variants associated with AIS [23–27]. Using whole exome sequencing, Buchan et al. [25] firstly analyzed rare variants in severe AIS cases and identified FBN1 and FBN2 as candidate genes for AIS, which were successfully replicated in two independent cohorts of patients from European ancestry and Han Chinese population, respectively. Through genetic linkage analyses combined with exome sequencing, Patten et al. [24] identified 3 rare missense variants in the POC5 that cosegregated with the disease in a large family with IS. Rare variants of POC5 were further replicated in an independent cohort of AIS patients, which added to the reliability of their findings [24]. Baschal et al. [27] performed exome sequencing in a four-generation family with IS and identified the variant p.Asn786Ser in the HSPG2 gene as a potential contributor to the phenotype. Enrichment of this variant was also confirmed in two independent cohorts of unrelated IS patients. In a recent study of Chinese population [23], a novel mutation of AKAP2 was observed in a family with AIS, which was believed to play a role in the etiology of AIS. However, the sample size of affected family members is small and there was a lack of functional analysis supporting the association of AKAP2 with AIS. In this study, we aimed to validate the relationship between AKAP2 and AIS in a large-scale general population.

## Methods

### Subjects

Medical records of patients who visited our clinic center for surgery between June 2012 and July 2016 were reviewed. The inclusion criteria were as follows: 1. Diagnosed as AIS; 2. With curve magnitude more than 45 degrees. The diagnosis of AIS was made through clinical and radiographic examinations by two experienced spine surgeons (X.L. and Z.Z.). All patients underwent MRI examination to exclude potential neurological defect. Baseline characteristics of the patients such as initial age and curve magnitude were recorded at their visit to our center. The curve magnitude was measured on the standing posteroanterior X-ray films using the Cobb method. The healthy participants were recruited through a community-based physical examinations program. All the control subjects were excluded to have scoliosis through Adam’s Forward Bend Test by an experienced spine surgeon (Q.Y.). Finally, a cohort of 1254 AIS patients and 1232 normal controls were included in our study, which was approved by the ethics committees of the local institution. All the subjects signed the informed consent before collection of the blood sample. Demographic data including birth place, gender, menarche age of female patients, curve magnitude, and BMI were recorded for each subject.

### Genotyping of rare variation

Genomic DNA was isolated from peripheral blood of the subjects using a blood extraction kit (Qiagen, Hilden, Germany) according to the manufacturer’s instructions. All DNA samples were genotyped for SNV c.2645A > C of AKAP2 (rs759883023) using allelic-specific multiple ligase detection reactions (LDR). The procedures of LDR experiment was carried out by the Shanghai Generay Biotech Co. Ltd. according to the standard protocol. The primers were designed using the genomic sequences in the GenBank (http://www.ncbi.nlm.nih.gov). To assess the validity of this procedure, 15% of samples were randomly selected and confirmed by direct DNA sequencing.

### RNA extraction and real-time qPCR

Blood samples were prospectively collected from AIS patients undergoing surgery in our clinic center and healthy controls who underwent physical examinations. Peripheral blood lymphocytes (PBMCs) were separated from blood using the Ficoll method. The blood samples were diluted with sterile PBS and poured onto the Ficoll solution. White blood cells were then collected using sterile pipette tips after centrifugation of the tubes at 2000 rpm for 20 min. The PBMCs were diluted with PBS and washed twice. Total RNA was isolated from PBMCs using TRIzol reagent (Invitrogen, Carlsbad, CA), which was reverse-transcribed using the PrimeScript RT Master Mix kit (Takara, Japan). Quantitative PCR analysis was performed using SYBR Premix Ex TaqTM II (Takara, Japan) in a 20 ul PCR mixture consisting of 10 ul of SYBR Premix Ex TaqTM II, 2.5 ul of template cDNA, and 0.4 uM of each PCR primer. Cycling conditions were as follows: 95 °C for 30 s, followed by 40 cycles at 95 °C for 5 s and 60 °C for 20 s. Samples were analyzed on the Roche LightCycler 480 II instrument (Roche Diagnostics, Mannheim, Germany). Glyceraldehyde-3-phosphate dehydrogenase (GAPDH) was used as the endogenous control gene for normalization. Relative mRNA expression was analyzed based on the ΔΔCt method. All amplifications were completed in triplicate. A mean value of threshold cycle (Ct) scores was calculated for the determination of relative expression levels. The specific primers are listed in Table 1.

**Table 1:** Primers for the genotypeing and qPCR assay

	Primer
rs759883023-TA	TTTTTTTTTTTTGCTTGCAGCCTGACTTAGCCCCTGA
rs759883023-TC	TTTTTTTTTTTTTTTGCTTGCAGCCTGACTTAGCCCCTGC
rs759883023-TR	AGAGGCTGCCGGAACCCAGCGGCCCTTTTTTTTT
*AKAP2*	Forward: TGCATTCTGCCGTGTTTATAGGTG
	Reverse: TGCCACTGACAGACCCTGTTTCC
*GAPDH*	Forward: GAGTCAACGGATTTGGTCGT
	Reverse: TTGATTTTGGAGGGATCTCG

### Genotyping of common variations in AKAP2

The GWAS database of AIS reported in our previous study was referred to for the genotyping information [16]. SNPs located within 5′ untranslated regions (UTR) and 3′ UTR of AKAP2 gene were selected with Haploview (v2.6). Among these variants, those encompassed in the Affymetrix Genome Wide Human SNP Array 6.0 (Affymetrix Inc., Santa Clara, California, USA) were further indentified, and the genotyping results were calculated for each SNP accordingly. Replication was performed in an independent cohort of 485 cases and 472 controls using Taqman probe genotyping assay as previously reported.

### Statistical analysis

SPSS version 17.0 (SPSS Inc., Chicago, USA) was used for the data analysis. Demographic data between the cases and controls were compared using the Student’s t test. PLINK version 1.90 (http://www.cog-genomics.org/plink2/) was used to calculate the association of each SNP with the disease by Cochran-Armitage trend test. The odds ratio (OR) values and 95% confidential intervals (CIs) were calculated on the basis of allele frequency table. Statistical significance was assumed at *P* < 0.05. Specifically, Bonferroni correction was applied to the analysis of GWAS data, with the significant level set at 0.0004 (0.05/116).

## Results

### Demographic data

Demographic data of the subjects were summarized in Table 2. For the patients, there were 1180 female and 74 male, with a mean age of 15.4 ± 3.5 years (range 10.8–18.7 years). The mean curve magnitude was 52.1 ± 12.5 degrees (range 27–63 degrees). 812 patients had Cobb angle more than 50 degrees and thus underwent surgical intervention. For female patients, the mean menarche age was 12.4 ± 1.9 years (range 10.3–14.1 years). All the patients and controls were Han population inhabiting along Yangtze River. There is a low possibility of ethnic heterogeneity among our subjects.

**Table 2:** Demographic data of the patients and the controls

	Patients (*n* = 1254)	Controls (*n* = 1232)	P
Age (years)	15.4 ± 3.5	22.5 ± 8.7	<0.001
Curve magnitude (degrees)	52.1 ± 12.5	N/A	N/A
Year post-menarche (years)	12.4 ± 1.9	N/A	N/A
Body mass index (kg/m^2^)	17.9 ± 3.8	22.3 ± 5.2	<0.001

N/A indicates not available

### Genotyping of rare variation and common variations

Our sequencing data showed that there was no case of mutation in c.2645A > C of AKAP2. All the patients and the controls present a genotype of AA.

The GWAS dataset was composed of 960 female AIS patients and 1499 controls. For the patients, the mean age was 14.3 ± 3.2 years (range 10.2–17.5 years). The mean curve magnitude was 38.5 ± 12.3 degrees (range 21–67 degrees). The allele frequencies of SNPs covering AKAP2 were summarized in Table 3. A total of 116 SNPs were analyzed, and none of these SNPs was found to have significantly different allele frequency between the cases and the controls. SNP rs7871428 was observed to have a *p* value of 0.02, which was further assessed in 485 cases and 472 controls. Specifically, the mean age of the patients was 13.8 ± 2.7 years (range 10.8–16.7 years). The mean curve magnitude was 41.3 ± 8.9 degrees (range 32–58 degrees). There was no significant differences regarding the minor allele frequency between the two groups (0.177 vs. 0.173, *p* = 0.82).

**Table 3:** The allele frequency of SNPs covering AKAP2

SNP	MA	MAF		P	OR (95% CI)
		Patients	Controls		
rs7022209	A	0.459	0.470	0.47	0.96 (0.85–1.08)
rs6477728	G	0.473	0.484	0.43	0.95 (0.85–1.07)
rs2769147	G	0.422	0.428	0.67	0.98 (0.87–1.10)
rs2795054	A	0.212	0.197	0.21	1.10 (0.95–1.26)
rs2795055	G	0.459	0.470	0.48	0.96 (0.86–1.08)
rs2182815	A	0.266	0.276	0.42	0.95 (0.83–1.08)
rs1887521	G	0.346	0.354	0.60	0.97 (0.86–1.09)
rs10980040	A	0.335	0.343	0.60	0.97 (0.86–1.09)
rs10980042	T	0.331	0.341	0.45	0.95 (0.85–1.08)
rs10980043	A	0.331	0.341	0.45	0.95 (0.85–1.08)
rs13289532	C	0.330	0.341	0.43	0.95 (0.84–1.08)
rs10759366	C	0.317	0.324	0.63	0.97 (0.86–1.10)
rs10759367	C	0.318	0.324	0.65	0.97 (0.86–1.10)
rs10980055	G	0.338	0.317	0.13	1.10 (0.97–1.24)
rs730973	A	0.346	0.330	0.23	1.08 (0.95–1.22)
rs1327785	A	0.336	0.346	0.46	0.96 (0.85–1.08)
rs10759369	A	0.319	0.324	0.71	0.98 (0.86–1.10)
rs12237683	T	0.336	0.346	0.48	0.96 (0.85–1.08)
rs10980064	C	0.318	0.324	0.62	0.97 (0.86–1.10)
rs3983508	C	0.346	0.330	0.25	1.07 (0.95–1.21)
rs4295727	T	0.328	0.314	0.30	1.07 (0.94–1.21)
rs2795058	A	0.273	0.285	0.37	0.94 (0.83–1.07)
rs7871857	C	0.147	0.142	0.59	1.05 (0.89–1.23)
rs12001151	C	0.156	0.140	0.13	1.13 (0.95–1.33)
rs2418050	A	0.483	0.476	0.63	1.03 (0.92–1.15)
rs7864423	A	0.149	0.135	0.16	1.13 (0.96–1.33)
rs7867595	A	0.149	0.134	0.16	1.12 (0.95–1.32)
rs1125416	A	0.147	0.142	0.57	1.05 (0.89–1.23)
rs2900498	C	0.100	0.091	0.34	1.10 (0.90–1.33)
rs875926	T	0.271	0.265	0.65	1.03 (0.91–1.17)
rs2769151	T	0.348	0.347	0.97	1.00 (0.89–1.13)
rs10816895	G	0.255	0.251	0.78	1.02 (0.89–1.16)
rs913361	T	0.256	0.267	0.41	0.95 (0.83–1.08)
rs17794822	A	0.181	0.178	0.80	1.02 (0.88–1.18)
rs10980086	C	0.276	0.268	0.51	1.04 (0.92–1.19)
rs10980091	C	0.468	0.454	0.34	1.06 (0.94–1.19)
rs12338023	G	0.193	0.188	0.70	1.03 (0.89–1.19)
rs2769143	C	0.227	0.228	0.95	1.00 (0.87–1.14)
rs2769145	G	0.087	0.084	0.72	1.04 (0.85–1.27)
rs17794870	T	0.178	0.177	0.88	1.01 (0.87–1.18)
rs9299178	G	0.192	0.194	0.86	0.99 (0.85–1.14)
rs2479311	A	0.238	0.239	0.99	1.00 (0.87–1.14)
rs10816902	C	0.193	0.193	0.94	0.99 (0.86–1.15)
rs10980097	C	0.192	0.188	0.67	1.03 (0.89–1.20)
rs7044189	C	0.364	0.351	0.35	1.06 (0.94–1.19)
rs13299632	C	0.281	0.286	0.70	0.98 (0.86–1.11)
rs7042517	T	0.439	0.446	0.65	0.97 (0.87–1.10)
rs2479314	G	0.306	0.316	0.44	0.95 (0.84–1.08)
rs16914570	T	0.112	0.104	0.40	1.08 (0.90–1.30)
rs2418060	A	0.444	0.449	0.70	0.98 (0.87–1.10)
rs2418061	T	0.441	0.447	0.66	0.97 (0.87–1.09)
rs7040294	T	0.444	0.449	0.73	0.98 (0.87–1.10)
rs1475433	T	0.445	0.447	0.92	0.99 (0.89–1.12)
rs16914573	A	0.111	0.102	0.35	1.09 (0.91–1.31)
rs13283300	C	0.205	0.200	0.70	1.03 (0.89–1.19)
rs9408826	A	0.156	0.140	0.13	1.14 (0.97–1.34)
rs12237174	A	0.252	0.239	0.31	1.07 (0.94–1.22)
rs10980109	G	0.149	0.139	0.29	1.09 (0.93–1.29)
rs7040787	A	0.278	0.275	0.79	1.02 (0.90–1.16)
rs4978856	T	0.239	0.249	0.40	0.94 (0.83–1.08)
rs7849107	A	0.251	0.249	0.89	1.01 (0.88–1.15)
rs2095094	C	0.079	0.091	0.16	0.86 (0.70–1.06)
rs1981035	A	0.238	0.249	0.37	0.94 (0.82–1.08)
rs1981036	A	0.240	0.248	0.56	0.96 (0.84–1.10)
rs877640	G	0.083	0.083	0.98	1.00 (0.81–1.23)
rs11999319	G	0.234	0.237	0.81	0.98 (0.86–1.13)
rs2277146	C	0.453	0.444	0.57	1.03 (0.92–1.16)
rs10120878	C	0.078	0.070	0.32	1.12 (0.90–1.39)
rs4978862	T	0.141	0.140	0.90	1.01 (0.86–1.19)
rs4978864	G	0.413	0.418	0.75	0.98 (0.87–1.10)
rs4978865	T	0.400	0.408	0.60	0.97 (0.86–1.09)
rs1930249	G	0.403	0.407	0.75	0.98 (0.87–1.10)
rs12683699	G	0.228	0.234	0.62	0.97 (0.84–1.11)
rs10739286	T	0.174	0.168	0.60	1.04 (0.89–1.21)
rs10980133	T	0.348	0.345	0.80	1.02 (0.90–1.15)
rs10816909	A	0.111	0.103	0.38	1.09 (0.90–1.31)
rs4978411	T	0.224	0.217	0.56	1.04 (0.91–1.20)
rs10217743	A	0.198	0.183	0.18	1.10 (0.95–1.28)
rs12000943	G	0.344	0.343	0.95	1.00 (0.89–1.13)
rs10739287	A	0.237	0.241	0.74	0.98 (0.85–1.12)
rs10816913	T	0.238	0.242	0.75	0.98 (0.86–1.12)
rs7864336	G	0.455	0.444	0.48	1.04 (0.93–1.17)
rs260193	A	0.367	0.358	0.51	1.04 (0.92–1.17)
rs7024673	A	0.235	0.250	0.23	0.92 (0.81–1.05)
rs260202	G	0.406	0.394	0.38	1.05 (0.94–1.19)
rs260221	C	0.449	0.437	0.41	1.05 (0.94–1.18)
rs10980164	T	0.381	0.394	0.38	0.95 (0.84–1.07)
rs10980169	A	0.063	0.078	0.05	0.80 (0.64–1.00)
rs2017393	G	0.378	0.387	0.51	0.96 (0.85–1.08)
rs2017392	G	0.378	0.387	0.53	0.96 (0.86–1.08)
rs2017219	C	0.389	0.395	0.68	0.98 (0.87–1.10)
rs541471	G	0.119	0.126	0.45	0.93 (0.78–1.11)
rs545955	C	0.456	0.437	0.21	1.08 (0.96–1.21)
rs552182	T	0.119	0.128	0.36	0.92 (0.77–1.10)
rs539817	T	0.448	0.433	0.32	1.06 (0.95–1.19)
rs475530	G	0.449	0.431	0.23	1.07 (0.96–1.21)
rs487013	C	0.485	0.464	0.14	1.09 (0.97–1.22)
rs491730	C	0.110	0.120	0.29	0.91 (0.76–1.09)
rs10759388	A	0.388	0.405	0.24	0.93 (0.83–1.05)
rs528811	T	0.365	0.382	0.23	0.93 (0.83–1.05)
rs10816924	A	0.182	0.165	0.12	1.13 (0.97–1.31)
rs10980210	G	0.286	0.307	0.10	0.90 (0.79–1.02)
rs7871428	G	0.199	0.173	0.02	1.19 (1.02–1.38)
rs4978870	A	0.214	0.221	0.61	0.96 (0.84–1.11)
rs17806457	A	0.205	0.208	0.81	0.98 (0.85–1.13)
rs2418071	C	0.076	0.071	0.48	1.08 (0.87–1.35)
rs10980217	C	0.201	0.203	0.87	0.99 (0.95–1.14)
rs3739455	G	0.173	0.176	0.80	0.98 (0.84–1.14)
rs16914814	C	0.177	0.182	0.67	0.97 (0.83–1.12)
rs1023180	A	0.114	0.109	0.58	1.05 (0.88–1.26)
rs10980229	A	0.417	0.408	0.54	1.04 (0.92–1.17)
rs10980231	C	0.154	0.151	0.76	1.03 (0.87–1.20)
rs4978877	G	0.501	0.491	0.48	1.04 (0.93–1.17)
rs10980234	G	0.287	0.279	0.56	1.04 (0.91–1.18)
rs10816929	G	0.308	0.305	0.84	1.01 (0.89–1.15)
rs1331310	C	0.229	0.228	0.95	1.01 (0.88–1.15)

### mRNA expression level of AKAP2 in blood

**Fig. 1:**
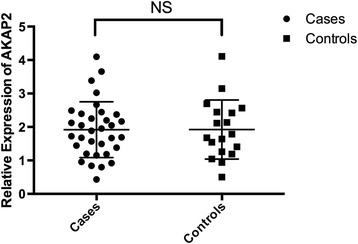
The mRNA expression of AKAP2 in blood analzyed for patients and age-matched controls. There was no significant difference between the patients (*n* = 33) and the controls (*n* = 20) regarding expression of AKAP2 in the blood (1.9 ± 0.8 vs. 1.8 ± 0.7, *p* = 0.66)

The expression level of AKAP2 in blood was successfully detected in 33 patients and 18 controls. The patients and the controls were matched in terms of age (15.2 ± 1.3 years vs. 15.8 ± 1.4 years, *p* = 0.13). As shown in Fig. 1, the mRNA expression of AKAP2 in patients was comparable with that in the controls (1.9 ± 0.8 vs. 1.8 ± 0.7, *p* = 0.66).

## Discussion

Aiming to validate the association of the rare variant in AKAP2 with the development AIS, we performed the genotyping of c.2645A > C in a cohort of 1254 AIS patients and 1232 normal controls, but found none case of mutation. Among a total of 116 common variants covering AKAP2, none was found to have significantly different allele frequency between the patients and the normal controls. Moreover, the mRNA expression of AKAP2 in the blood was comparable between the AIS patients and the controls. Obviously, the previously reported association of AKAP2 with the susceptibility of AIS in Chinese population was not replicated in the present study. It is noteworthy that there were different study designs between our study using population-based samples and the previous study of Li et al. [23] using familial patients. In their study, a mutation of c.2645A > C was detected through whole exome sequencing in the 5 affected members with AIS, 2 of whom were found to have remarkably decreased expression level of AKAP2. In this study, we recruited a large number of sporadic patients and normal controls which we believed were powerful enough to detect the effect sizes as reported by the study of Li et al. [23]. Herein, it is likely that the mutation of AKAP2 may play a role in the development of familial AIS, while its role in the general AIS population should be further validated.

Panza et al. [28] reported a balanced de novo translocation t(7;9)(p14.1;q31.3) that disrupts AKAP2 gene on chromosome 9. In such case of rare translocation linked to the disease, genes on chromosome 7 may demonstrate stronger association with AIS. Therefore, we cannot rule out that there could also be such event in AIS patients, since previous linkage study has reported evidence suggestive of linkage in Chromosome 7 [29]. Pinpointing the susceptible genes located at 7p14.1 may be an interesting topic worthy of further investigation.

Studying the genetics of AIS is usually difficult since there is a high degree of genetic heterogenicity among patients [7]. AIS can be a potential presence of many different genetic defects, all of which may eventually lead to the same clinical phenotype recognized as spinal curves. It is well documented that there is a lack of replication on most of the previously reported predisposition genes of AIS except for LBX1, MMP3 and IL6 [30–33]. Recently, mechanism underlying the role of LBX1 in the development of AIS was illustrated by the functional characterization of the most significantly associated SNP which presents a novel pathological feature of LBX1 in body axis deformation [34]. Herein, without strong evidence produced through functional experiment, research merely based on simple association analysis cannot be sound enough to establish a reproducible result. Of note, the primary limitation of the study reported by Li et al. [23] lies in that the relationship between mutation of c.2645A > C and the expression of AKAP2 was not validated through in-vivo cellular experiment such as luciferase assay. Taken together, the function of AKAP2 as a predisposition gene of AIS should be cautiously interpreted.

## Conclusions

Our large-scale replication study of the variants in AKAP2 gene did not support its association with the susceptibility of AIS in the Chinese population. In future study, functional studies of the previously reported rare variant are warranted to clarify whether the variant can regulate the expression of AKAP2. The whole AKAP2 gene can be sequenced in larger AIS cohorts to identify potentially missing mutations.

## Abbreviations

AIS: Adolescent idiopathic scoliosis
AKAP2: A-Kinase Anchoring Protein 2
CDH13: Cadherin 13
ESR1: Estrogen receptor 1
FBN1: Fibrillin 1
FBN2: Fibrillin 2
GWAS: Genome-wide association study
NTF3: Neurotrophin 3
PAX1: Paired box 1
POC5: POC5 centriolar protein
SNP: Single nucleotide polymorphism
SNV: Single nucleotide variant
UTR: Untranslated regions
